# Efficient transgene-free multiplexed germline editing via viral delivery of an engineered TnpB

**DOI:** 10.64898/2026.01.23.700382

**Published:** 2026-02-25

**Authors:** Trevor Weiss, Maris Kamalu, Honglue Shi, Gabriel Wirnowski, Alice Ingelsson, Jasmine Amerasekera, Kamakshi Vohra, Marena I. Trinidad, Zheng Li, Emily Freitas, Noah Steinmetz, Charlie Ambrose, Kerry Chen, Jennifer A. Doudna, Steven E. Jacobsen

**Affiliations:** 1 Department of Molecular, Cell and Developmental Biology, University of California at Los Angeles, Los Angeles, CA, USA, 90095; 2 Innovative Genomics Institute, University of California, Berkeley, CA, USA, 94720; 3 Howard Hughes Medical Institute, University of California, Berkeley, CA, USA, 94720; 4 California Institute for Quantitative Biosciences (QB3), University of California, Berkeley, Berkeley, CA, USA, 94720; 5 University of California, Berkeley-University of California, San Francisco Graduate Program in Bioengineering, University of California, Berkeley, Berkeley, CA, USA, 94720; 6 Howard Hughes Medical Institute, University of California at Los Angeles, Los Angeles, CA, USA, 90095

## Abstract

Virus-induced genome editing (VIGE) using compact RNA-guided endonucleases is a transformational new approach in plant biotechnology, enabling tissue-culture-independent and transgene-free genome editing (Hu et al. 2025; Liu et al. 2025; Weiss et al. 2025). We recently established a VIGE approach for heritable editing at single loci in *Arabidopsis* by delivering the compact genome editor ISYmu1 TnpB (Ymu1) and its guide RNA (gRNA) via Tobacco Rattle Virus (TRV) (Weiss et al. 2025). Here, we greatly improved this approach by devising a multiple gRNA expression system and by utilizing an engineered high-activity Ymu1 variant (Ymu1-WFR) (Zhou et al. 2026) to develop an efficient multiplexed genome editing platform.

TRV is a bipartite RNA virus composed of RNA1 and RNA2. To evaluate TRV-mediated multiplexing capabilities, we co-delivered RNA1 with two RNA2 vectors encoding either *AtPDS3* gRNA12 or *AtCHLl1* gRNA4 to *Arabidopsis* (Figure S1A), two gRNAs with high activity (Weiss et al. 2025). Amplicon sequencing (amp-seq) revealed editing almost exclusively at one target site or the other (Figure S1B), suggesting viral superinfection exclusion (Perdoncini Carvalho et al. 2022). We therefore sought to develop a system in which both gRNAs could be expressed on a single RNA2 vector.

First, to find the optimal gRNA structure we identified the precise omega RNA (ωRNA) sequence via small RNA sequencing (RNA-seq) in *E. coli*, and found it to be 127-nucleotides (nt) in length (Figures S2A, S2B). In addition, we tested various ωRNA lengths and found that 127-nt gave the highest editing in protoplasts (Figures S2C, S2D). Using the 127-nt ωRNA, we tested multiplexed arrays featuring tRNA, HDV, HDV-HH, or a repeat as gRNA processing elements using an *Arabidopsis* protoplast assay (Figure S2F). Amp-seq analysis showed that while all designs enabled editing, HDV-based designs performed best at simultaneously editing both sites in protoplasts (Figure S2G). Furthermore, polymerase chain reaction (PCR) using primers spanning both sites suggested the occurrence of large deletions between the two target sites (Figure S2H). These experiments identified the HDV and HDV-HH designs as the top performing multiplexing arrays, and that Ymu1 TnpB can generate large deletions between two targeted sites.

To minimize RNA2 cargo size, the HDV multiplex array was selected for TRV-mediated multiplexed editing *in planta*. Initially, we designed two RNA2 vectors targeting *AtCHLl1* (gRNA4) and *AtPDS3* (gRNA12), incorporating a tRNA^Ileu^ mobility sequence at the 3’ end of the cargo to facilitate systemic movement and heritability (Figure S3A). gRNA4 targets the gene body of *AtCHLl1* (biallelic edits create yellow tissue sectors) and gRNA12 targets the promoter region upstream of the *AtPDS3* transcription start site (no visible phenotype). After delivering TRV vectors, we did not observe any phenotypic evidence of editing. Suspecting inefficient mobility or processing of the RNA2 cargo, we tested three additional constructs containing a tRNA^Ileu^ downstream of each HDV ribozyme (Figure 1A). After TRV delivery, yellow sectors appeared on leaves for all three vectors, indicating biallelic edits at *AtCHLl1* (Figure 1B). Yellow sectored plants infected with vectors pTW2278 and pTW2279 displayed average editing efficiencies of 25.6% for *AtCHLl1* and 30.2% for *AtPDS3* (Figure 1C), and those infected with pTW2498 showed 31.3% for *AtCHLl1* gRNA4 and 5.2% for *AtCHLl1* gRNA6 (Figure 1D).

We recently engineered a highly active Ymu1 variant (Ymu1-WFR) (Zhou et al. 2026). To evaluate Ymu1-WFR efficiency using TRV, we targeted three published *AtCHLl1* target sites: gRNA4, gRNA6, and gRNA9 (Weiss et al. 2025). Infected plants showed a strong yellow phenotype for all three gRNAs (Figure 1E). Amp-seq revealed average editing efficiencies of 18.6%, 14.7%, and 9.8%, respectively, much higher (up to 9.8-fold) than wild type (WT) Ymu1 (Weiss et al. 2025) (Figure 1F).

To assess the impact of the WFR variant on multiplexed editing efficiency, we replaced the WT Ymu1 sequence in pTW2278, pTW2279, and pTW2498 with Ymu1-WFR (Figure S3B). Following TRV delivery, we observed much more pronounced phenotypic evidence of editing than with WT Ymu1 (Figure 1G compared with Figure 1B). Amp-seq of yellow sectored plants confirmed enhanced editing: pTW2657 and pTW2658 averaged 48.9% for *AtCHLl1* (gRNA4) and 41.3% for *AtPDS3* (gRNA12) (Figure 1H), while pTW2655 averaged 45.3% and 28.1% at the two *AtCHLl1* sites (Figure 1I). Consistent with the protoplast result (Figure S2H), PCR analysis using primers spanning the *AtCHLl1* gRNA4 and gRNA6 sites revealed large deletions (Figures S3C, S3D).

To characterize germline transmission of multiplexed *AtPDS3* gRNA12 and *AtCHLl1* gRNA4 edits, we selected two plants infected with WT Ymu1 (pTW2278_17 and pTW2278_54) and two plants infected with Ymu1-WFR (pTW2658_5 and pTW2658_80). We observed yellow progeny at frequencies of 4.7% and 1.7% using WT Ymu1, and 6.1% and 12.9% with Ymu1-WFR (Figure 1J, S3E, Table S1). Among the yellow seedlings, the majority of them harbored biallelic edits at both loci (Figure 1J, S3F). Additionally, targeting *AtCHLl1* with two gRNAs, gRNA4 and gRNA6 (using pTW2655), resulted in 8.2% and 24.1% yellow seedlings, with 36/158 (22.8%) of the progeny harboring homozygous large deletions between the two targets (Figures 1J, S3G-I). These data demonstrate that TRV effectively delivers Ymu1-WFR and multiple gRNAs for efficient multiplexed germline editing, that biallelic editing at one locus is highly predictive of biallelic editing at the second target site, and that co-targeting the same gene gave progeny with large deletions between the two target sites.

By optimizing the gRNA array design, and incorporating the highly active engineered Ymu1-WFR variant, we developed an efficient multiplexed editing platform that bypasses the need for transgenesis. Given the broad host range of TRV, we anticipate this approach will be adaptable to many crop species, for example tomato where germline editing has already been demonstrated (Liu et al. 2025). Additionally, the ability to generate large deletions should expand this system’s utility for regulatory element engineering. Finally, this multiplexed system may enable the study of embryonic lethal genes by utilizing *AtCHLl1* as a visual marker; the yellow somatic sectors should facilitate the identification of tissue harboring biallelic knockout of a gene of interest.

## Supplementary Material

Supplement 1

## Figures and Tables

**Figure 1:**
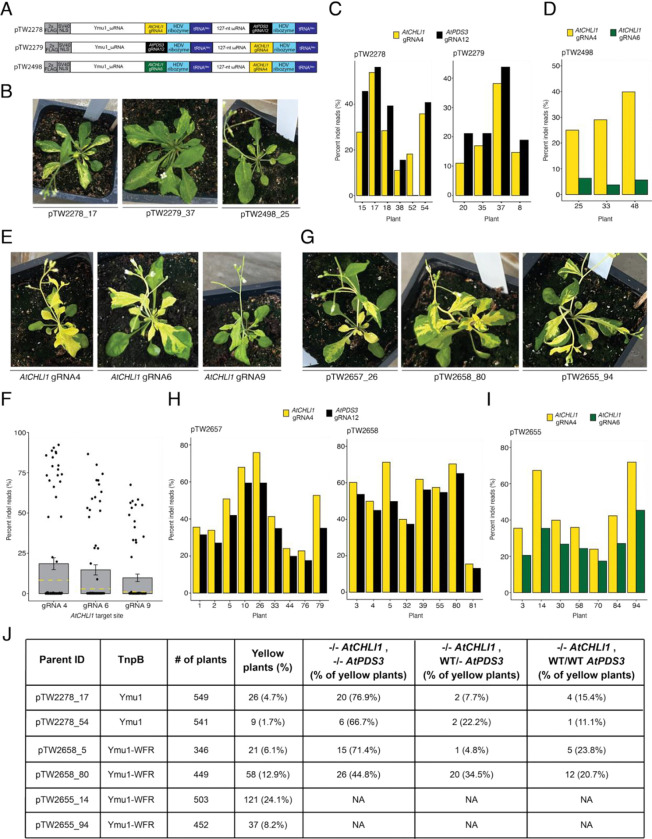
Heritable and transgene-free multiplexed genome editing in *Arabidopsis* via viral delivery of Ymu1. **(A)** Schematic representation of the cargo being expressed by the PEBV promoter in TRV RNA2. *AtCHLl1* gRNA4 (yellow), *AtPDS3 gRNA12* (black), and *AtCHLl1* gRNA6 (green) were used in the multiplex TRV experiments. The plasmid ID is listed to the left of each construct. **(B)** Yellow sector phenotype observed from plants infected with the TRV vectors from panel A. The plant ID (construct_plant) is listed below each picture. **(C and D)** Editing efficiency (y-axis) of plants displaying the yellow sector phenotype (x-axis) infected with TRV vectors pTW2278, pTW2279 and pTW2498. **(E)** Yellow sector phenotype observed from plants infected with TRV vectors expressing Ymu1-WFR targeting *AtCHLl1* (gRNA4, gRNA6, and gRNA9). The site being targeted is listed below each picture. **(F)** Editing efficiency (y-axis) of plants infected with TRV vectors expressing Ymu1-WFR targeting *AtCHLl1* (x-axis). Each dot represents an individual plant. The yellow dashed line on each bar indicates the average editing efficiency previously reported (Weiss et al. 2025). **(G)** Yellow sector phenotype observed from plants infected with TRV vectors expressing Ymu1-WFR targeting *AtCHLl1* gRNA4 and *AtPDS3* gRNA12 (pTW2657 and pTW2658) or *AtCHLl1* gRNA4 and *AtCHLl1* gRNA6 (pTW2655). The plant ID (construct_plant) is listed below each picture. **(H and I)** Editing efficiency (y-axis) of plants displaying the yellow sector phenotype (x-axis) infected with TRV vectors pTW2657, pTW2658 and pTW2655. (**J**) Heritability data from plant ID pTW2278_17, pTW2278_54, pTW2658_5, pTW2658_80, pTW2655_14, and pTW2655_94.

## Data Availability

Amp-seq data is accessible at NCBI Sequence Read Archive BioProject PRJNA1427739. RNA-seq data is available at GEO accession: GSE316183.
